# Supplementary material for: Monoclonal neutralizing antibodies elicited by infection with Kaposi sarcoma-associated herpesvirus reveal critical sites of vulnerability on gH/gL
Source: PLoS Pathog. 2026 Jan 7;22(1):e1013772. doi: 10.1371/journal.ppat.1013772 (PMC12795454; doi:10.1371/journal.ppat.1013772)
Supplement: S1 Table — (DOCX) [file ppat.1013772.s001.docx]

S1 Table: Ontogenies of gH/gL specific antibodies.

| mAb | Participant ID | Heavy Chain V-Gene | CDRH3 | Genbank Accession | Light Chain V-Gene | CDRL3 | Genbank Accession |
| --- | --- | --- | --- | --- | --- | --- | --- |
| MLKH1 | U035-22-MO | IGHV1-69*01 | CVRESGVYYDTSGYYYSDGFDIW | PV035824 | IGLV7-43*01 | CLLFSGGARVF | PV035834 |
| MLKH2 | U035-22-MO | IGHV4-61*09 | CARDRRNNRWPVYVFDIW | PV035825 | IGKV3-20*01 | CQQYGNSPIFTF | PV035835 |
| MLKH3 | U035-22-MO | IGHV1-69*11 | CAISEGGYTYDSGSYIW | PV035826 | IGKV1D-12*01 | CQQANSFPYTF | PV035836 |
| MLKH4 | U035-22-MO | IGHV3-66*02 | CARAFRSYGAFDLW | PV035827 | IGKV4-1*03 | CQQYYNTPWTF | PV035837 |
| MLKH5 | U035-22-MO | IGHV1-69*06 | CARESGVYYDTSGYYYSDTFDLW | PV035828 | IGLV7-43*01 | CLLFSGGARVF | PV035838 |
| MLKH6 | U035-22-MO | IGHV4-61*09 | CARGRRTTGIAAAVCDSW | PV035829 | IGKV3-15*02 | CQQYNNWPPWTF | PV035839 |
| MLKH7 | U035-22-MO | IGHV4-61*11 | CARGRVAGGHCCVFDPW | PV035830 | IGKV3-15*02 | CQHYHNWSLTF | PV035840 |
| MLKH8 | U035-22-MO | IGHV3-48*03 | CVRGSFGDFYYNYYYYMDVW | PV035831 | IGLV2-11*01 | CCSYAGRYNVVF | PV035841 |
| MLKH9 | U035-22-MO | IGHV4-61*09 | CARGRVAAGKRALFDPW | PV035832 | IGKV3-15*02 | CQQYNNWPPGTF | PV035842 |
| MLKH10 | U035-22-MO | IGHV3-20*04 | CARVQGSGSYNNFDYW | PV035833 | IGKV4-1*03 | CQQYYNSPRF | PV035843 |
| MLKH11 | U035-67-MO | IGHV1-8*01 | CARGLPSDLSSTYYPFW | PX122841 | IGLV8-61*01 | CLLYMGGGVWEF | PX122842 |
| MLKH12 | U035-67-MO | IGHV3-48*01 | CARDKVATTTTSPLYFYYYYGMDVW | PX122843 | IGKV1-12*01 | CQQANSFPLTF | PX122844 |
