# Supplementary material for: Monoclonal neutralizing antibodies elicited by infection with Kaposi sarcoma-associated herpesvirus reveal critical sites of vulnerability on gH/gL
Source: PLoS Pathog. 2026 Jan 7;22(1):e1013772. doi: 10.1371/journal.ppat.1013772 (PMC12795454; doi:10.1371/journal.ppat.1013772)
Supplement: S2 Table — (DOCX) [file ppat.1013772.s002.docx]

S2 Table: Properties of gH/gL specific antibodies.

| mAb | Binding affinity (kD)  nM | Cluster | Neutralizing potency IC_50_ µg/ml | Inhibits syncytia formation | Blocks EphA2 binding |
| --- | --- | --- | --- | --- | --- |
| MLKH1 | 0.24 | 1 | 0.04 | Yes | Yes |
| MLKH2 | 1.01 | 3 | Non-neutralizing | Yes | No |
| MLKH3 | 1.14 | 4 | 0.08 | Yes | No |
| MLKH4 | 60.7 | 2 | Non-neutralizing | No | No |
| MLKH5 | 0.41 | 1 | 0.02 | Yes | Yes |
| MLKH6 | 0.4 | 2 | 0.06 | Yes | No |
| MLKH7 | 0.58 | 2 | 1.01 | Yes | No |
| MLKH8 | 759 | 4 | Non-neutralizing | No | No |
| MLKH9 | 441 | 2 | Non-neutralizing | Yes | No |
| MLKH10 | 165 | 5 | Non-neutralizing | Yes | No |
| MLKH11 | 0.46 | 2 | 0.25 | Yes | No |
| MLKH12 | 5.25 | 2/3 | 1.66 | Yes | No |
