# Supplementary figures and images for: Monoclonal neutralizing antibodies elicited by infection with Kaposi sarcoma-associated herpesvirus reveal critical sites of vulnerability on gH/gL
Source: PLoS Pathog. 2026 Jan 7;22(1):e1013772. doi: 10.1371/journal.ppat.1013772 (PMC12795454; doi:10.1371/journal.ppat.1013772)

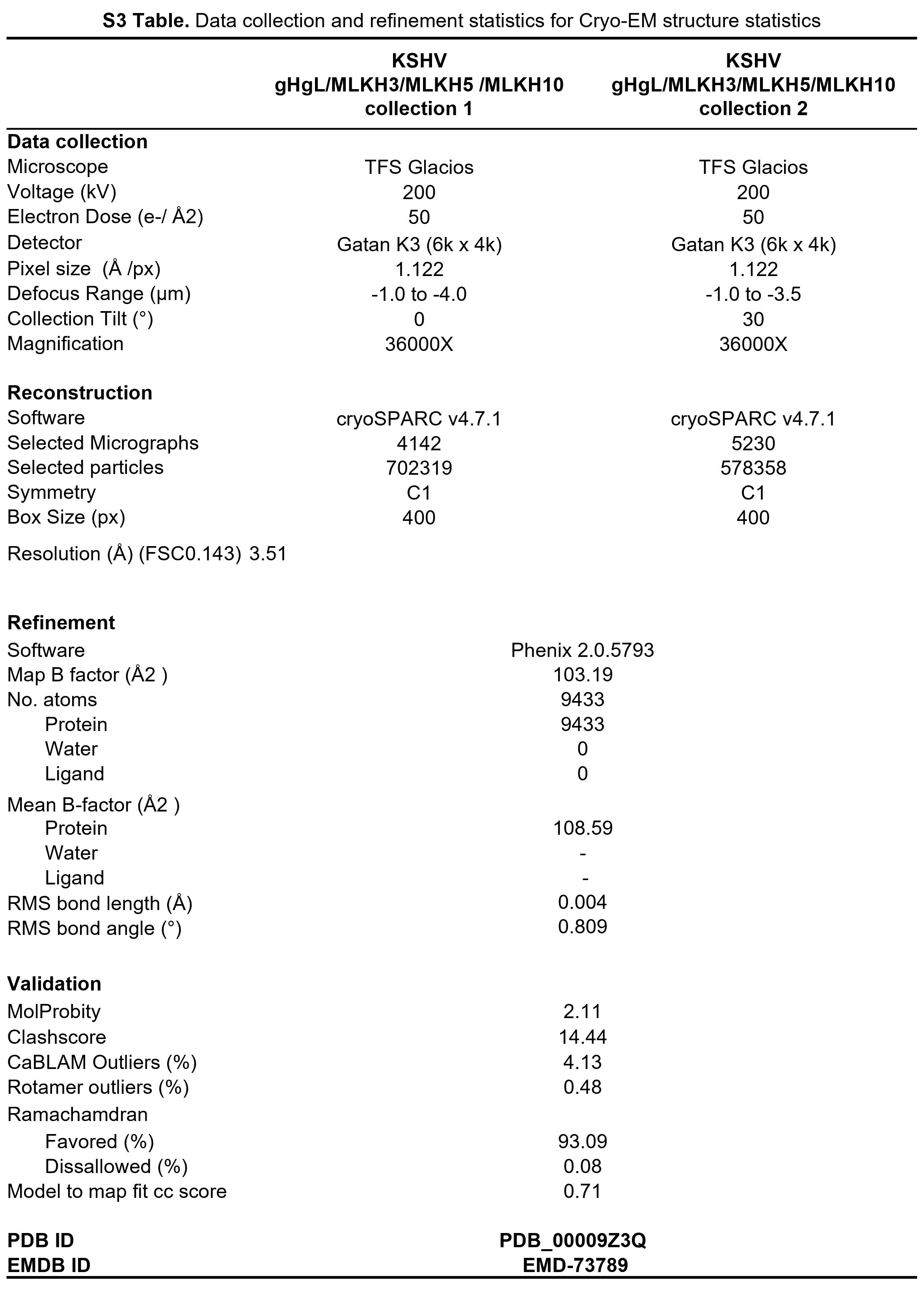

Supplement: S3 Table — (TIF) [file ppat.1013772.s003.tif]

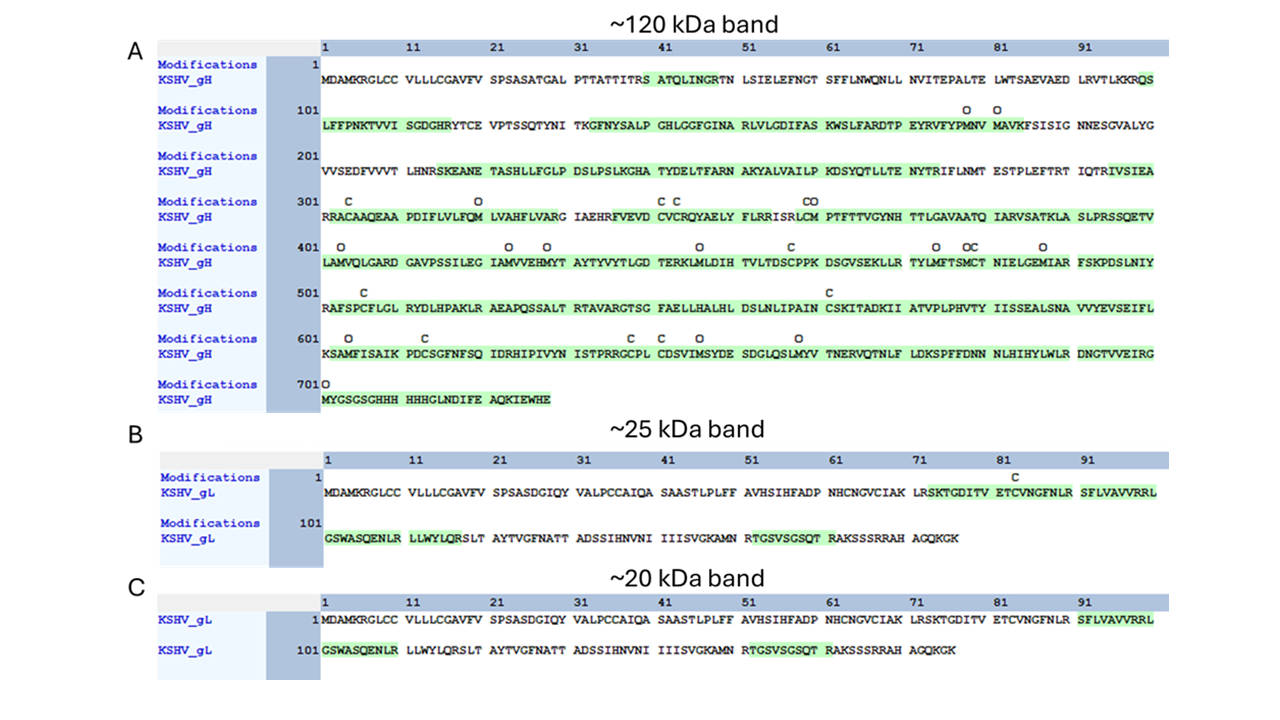

Supplement: S1 Fig — The indicated bands from the right-hand lane of the SDS-PAGE gel in Fig 1D were excised an analyzed by proteolytic cleavage followed by mass spectrometry. The peptides identified in the mass spectra that map to gH (A) and gL (B and C) are highlighted on the protein sequences in green. Cysteine residues with carbamidomethylation and oxidized methionine residues are indicated with C and O, respectively. (TIF) [file ppat.1013772.s004.tif]

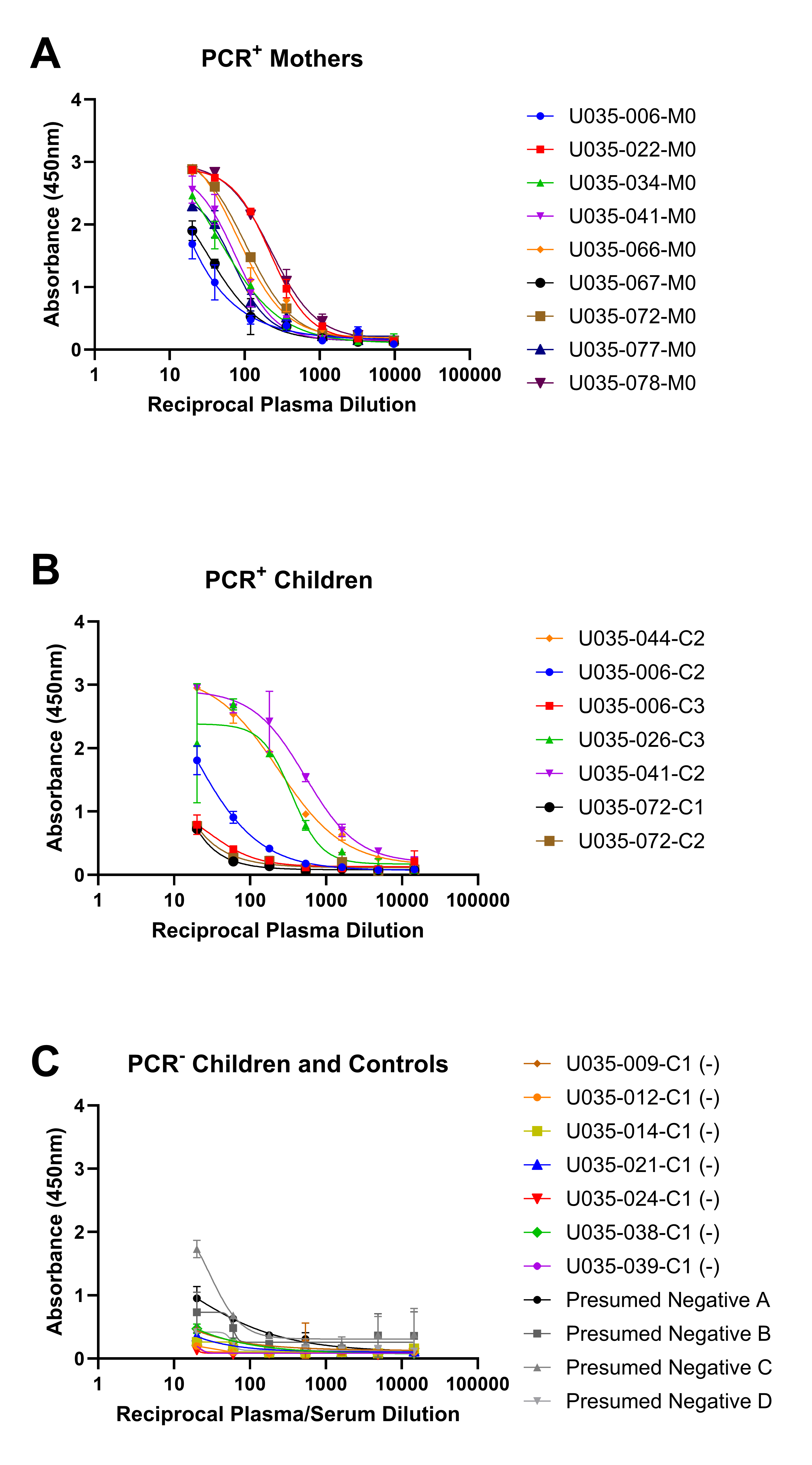

Supplement: S2 Fig — Source data from Fig 2A. (A) Plasma from mothers who tested positive for KSHV DNA (+), (B) Plasma from household children who tested positive for KSHV DNA (+), and (C) plasma from household children who tested negative for KSHV DNA (-), as well as serum from presumed KSHV-negative donors from the Seattle-area were serially diluted and tested for binding to gH/gL by ELISA as indicated. (TIF) [file ppat.1013772.s005.tif]

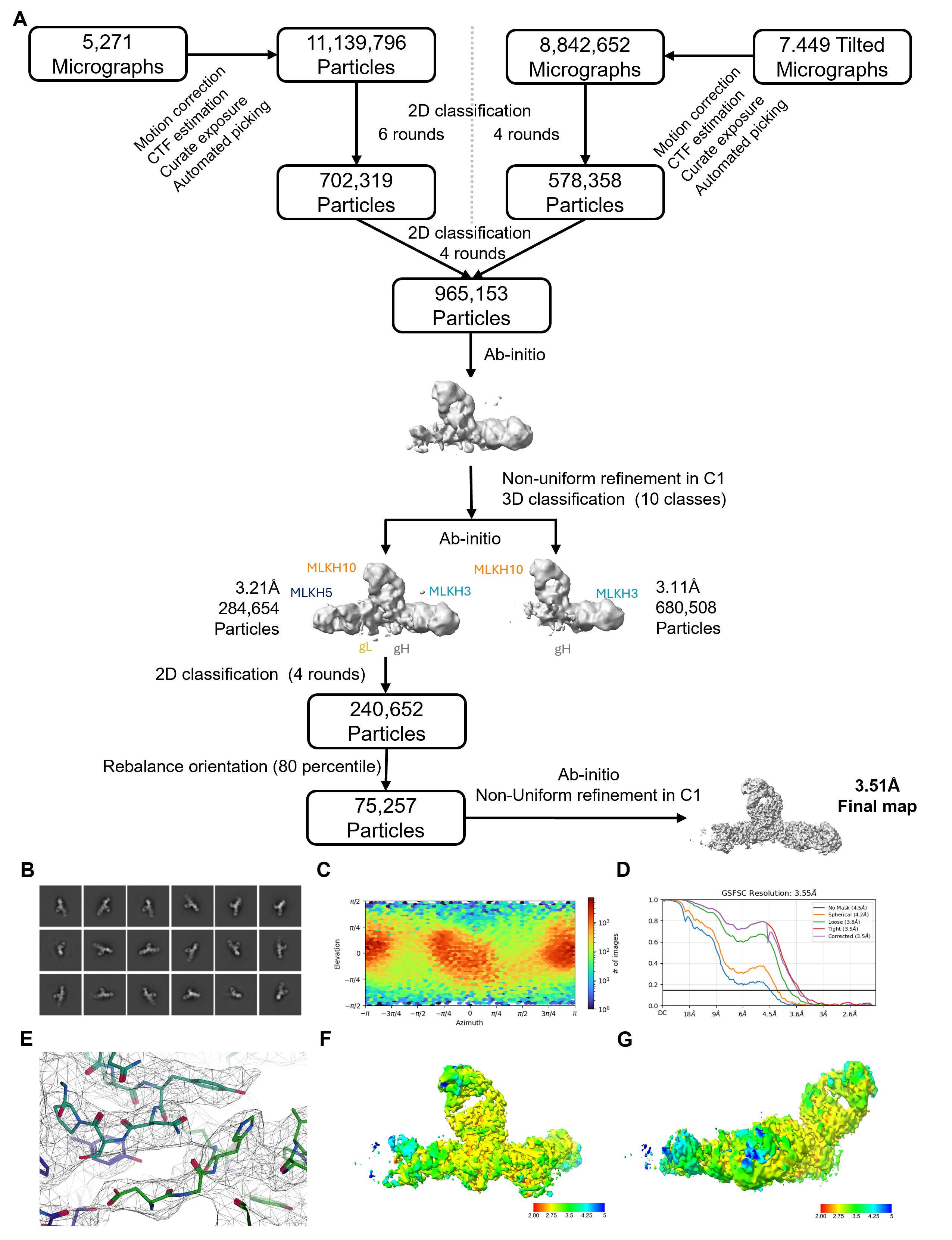

Supplement: S3 Fig — (A) Data processing workflow (B) 2D classification. (C) View direction distribution plot of particles in 3D reconstruction. (D) Resolution estimation, CryoSPARC GSFSC. (E) Model fit image to 3D reconstruction, focused on MLKH10 and gH interface. (F-G) Local resolution map emphasizing the MLKH10 interface (F) and a 90° rotated view for the MLKH3 interface (G). (TIF) [file ppat.1013772.s006.tif]

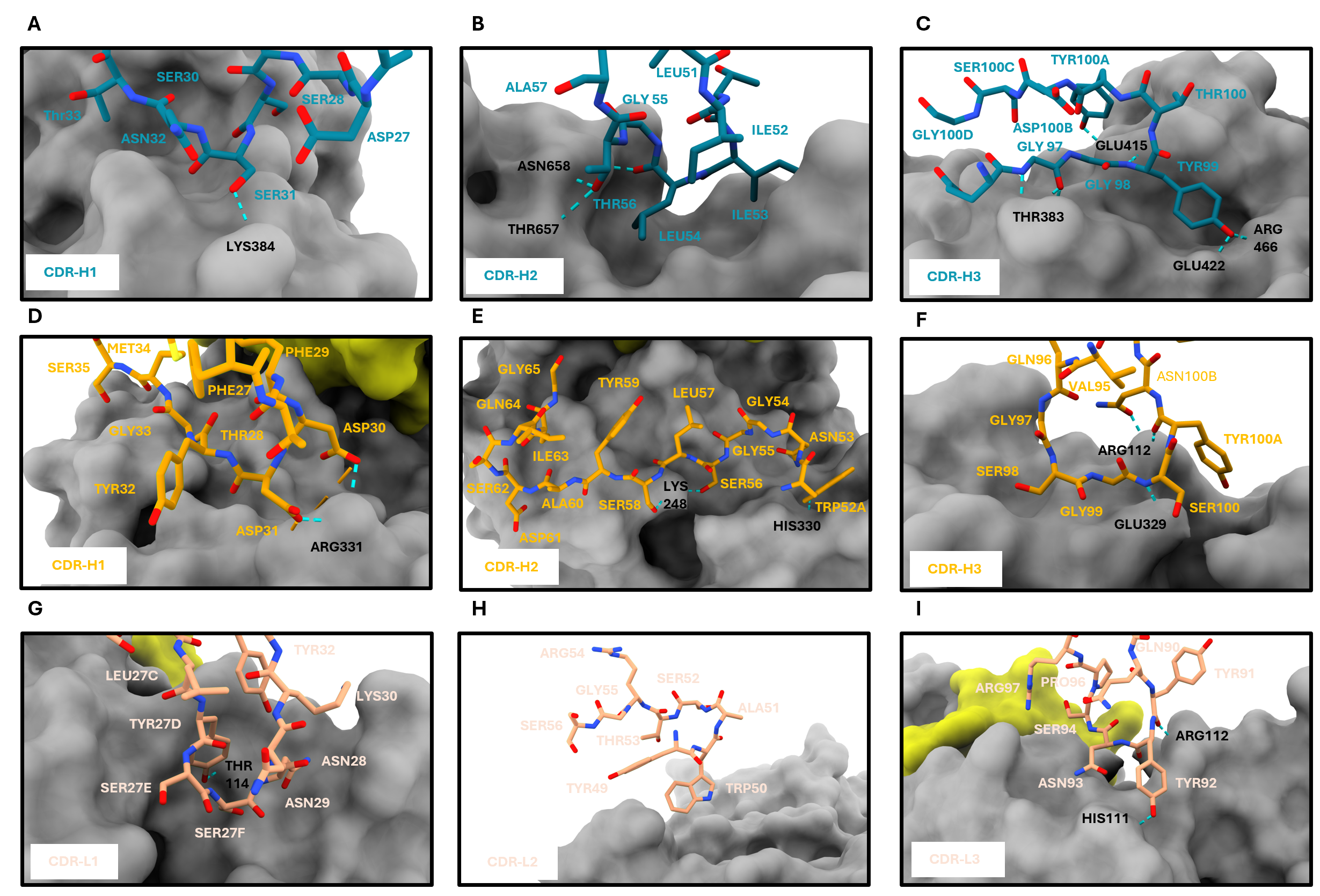

Supplement: S4 Fig — (A-C) MLKH3 CDR-H1, H2 and H3 are shown in teal with gH in grey surface as indicated. (D-F) MLKH10 CRD-H1, H2 and H3 shown in orange with gH in grey surface and gL in yellow surface. (G-I) MLKH10 CRDR-L1, L2 and L3 shown in salmon with gH in grey surface and gL in yellow surface. In all panels hydrogen bonds are shown as cyan dashed lines and salt bridges as yellow dashed lines. (TIF) [file ppat.1013772.s007.tif]
