# Supplementary material for: Monoclonal neutralizing antibodies elicited by infection with Kaposi sarcoma-associated herpesvirus reveal critical sites of vulnerability on gH/gL
Source: PLoS Pathog. 2026 Jan 7;22(1):e1013772. doi: 10.1371/journal.ppat.1013772 (PMC12795454; doi:10.1371/journal.ppat.1013772)
Supplement: Attachment — Submitted filename: Rebuttal.pdf [file ppat.1013772.s010.pdf]

Response to Reviewer Comments

We thank the reviewers for their careful consideration of our manuscript. We have included their comments below. Our responses appear in **bold blue** font. We note that any references to specific lines on the manuscript file refer to the marked up version, not the clean version.

We also would like to highlight that during the review and revision process we have been working to obtain higher resolution structures of the mAbs we describe in our manuscript in complex with gH/gL. We include a new cryoEM structure of the MLKH3 and MLKH10 mAbs bound to gH/gL in the revised submission. This appears as Figure 8 and associated supplementary materials S5-S7.

Reviewers' Comments:

Reviewer's Responses to Questions

Part I - Summary
Please use this section to discuss strengths/weaknesses of study, novelty/significance, general execution and scholarship.

Reviewer #1: Wu et al. report a characterization of a panel of human antibodies targeting the gH/gL glycoprotein complex of KSHV, an oncogenic human virus. This work addresses an important gap in knowledge, as previous studies have been limited to the use of polyclonal sera (animal or human). In this manuscript, a dozen human mAbs, isolated from KSHV-infected individuals, are characterized in terms of their binding affinity and their potency in neutralizing the virus or blocking cell-cell fusion. Low-resolution reconstructions of five neutralizing mAbs bound to gH/gL, obtained from negative-stain EM images, are provided, revealing vulnerable sites on the viral glycoprotein. The data are, in general, well-presented, and the experiments are well-designed and executed, with some room for improvement as suggested in the major and minor points sections below.

Reviewer #2: Wan et al. report highly interesting findings on the discovery of neutralizin antibodies to KSHV gH/gL and distinct antigenic sites on KSHV gH/gL that serve as targets for these neutralizing antibodies. The authors used a cohort of Ugandan mothers and children to determine seroreactivity to KSHV gH/gL and to isolate neutralizing antibodies through antigen-specific B cell sorting. The finding that most subjects in the cohort show high reactivity to gH/gL is interesting, there is a surprisingly high number of potent neutralizers among the mothers and also their children, which conflicts with the literature.

This part of the study raises some questions that are not properly discussed. Also, the KSHV PCR is not properly explained (RT-PCR?). Showing some true negative controls would be really helpful here, and the neutralization assay might suffer from complement effects if the methods are as stated in the manuscript and did not incorporate a heat inactivation step.

The identification of 12 binding and neutralizing antibodies is of high value to the community.
In their structural and elegant mutual competition studies, in addition to the EphA2 receptor binding site, the authors identified two additional antigenic sites as targets of neutralizing antibodies. Through electron microscopy analysis, the authors identified the rough location of the antigenic sites.
Sequence analysis showed that some antibodies from one individual were clonally related or shared VH/VL gene usage, which may suggest reproducible pathways of antibody development.

The functional charactization of the antibodies is interesting, in particular the observed differences between neutralizing acitivty in an epithelial cell infection assay, a cell-cell-based fusion assay, and an infection assay with a B cell line. The latter findings suffer from some overinterpratation.

Whether the structural nsEM data is sound/of sufficient quality or needs further experimental confirmation through e.g. mutagenesis should be decided by an expert reviewer with deeper understanding of these technical aspects.

Part II – Major Issues: Key Experiments Required for Acceptance
Please use this section to detail the key new experiments or modifications of existing experiments that should be <u>absolutely</u> required to validate study conclusions.

Generally, there should be no more than 3 such required experiments or major modifications for a "Major Revision" recommendation. If more than 3 experiments are necessary to validate the study conclusions, then you are encouraged to recommend "Reject".

Reviewer #1: • Lines 119-22 and Fig 1D and 1E: the 3D reconstruction of gH/gL does not seem convincing. The ribbon model (1E) should be placed inside the map (1D), and the correlation coefficient (cc) should be provided. Otherwise, the panels D and E may be the best removed, as they do not add to the story and indeed gH/gL may be too small to obtain good 3D reconstructions. It would also be helpful if gH and gL were represented in more contrasted colors (thru the manuscript, figures 6D, 7 included).

We have removed panels D and E from Figure 1 since we are able to obtain more meaningful reconstructions in complex with mabs in Figs. 6, 7 and 8. We have also changed the color scheme of gH/gL in Figures 6 and 7 as per the reviewer's suggestion.

• CC values for fitting of the Fabs and gH/gL into maps should be provided for representations on Fig 6B, 6C and Fig 7.

We provide the CC value for the new cryo-EM structure in Table S7. However, a CC score is not a standard value in negative stain electron microscopy (nsEM). The score is calculated in real space by comparing the density values of the experimental map with a simulated map generated from the atomic model. Model parameters such as coordinates, occupancies, atomic displacement parameters, and chemical atom types are required for this calculation and are extracted from the input model file. The unit cell parameters are also required.

Since the model used was not refined against a negative staining map—due to the lower resolution limit of nsEM (typically around 20 Å)—and because the model was not generated from our own experiment but instead obtained from the PDB, the CC values won't be accurately calculated compared to those obtained from high-resolution techniques like cryo-EM.

Nevertheless, we calculated them for the reviewer's information, and the cc box values are as follows (consider good at > 0.6):

- **MLKH3–MLKH5–MLKH10/gHgL complex: 0.75**
- **MLKH6–MLKH5–MLKH10/gHgL complex: 0.72**
- **MLKH12–MLKH5–MLKH10/gHgL complex: 0.70**
- **MLKH1/gHgL complex: 0.70**
- **MLKH5/gHgL complex: 0.81**

• Hahn et al. have shown that KSHV gH can be expressed and secreted without gL, which typically plays the role of a chaperone and is essential for gH expression in other herpesviruses (PMID: 18945775). In this manuscript, the gH/gL complex was purified via an affinity tag at the C-terminus of gH, which likely resulted in a SEC peak containing gH alone

as well as the gH/gL complex. Thus, the statement in lines 125-126, "gH/gL is highly pure," is questionable. Could the authors comment on that?

The reviewer raises an excellent point and we believe they are correct. In the revised manuscript we analyzed gH/gL fractions from gH/gL purified by size exclusion chromatography (Fig. 1B-C). We show that the gH/gL preparation elutes as a single peak, with no difference in the amount of gL present at the front or tail of the peak. We modified the text as follows on lines 123-130 " KSHV gH can express without gL[50]. Therefore, the purified protein could be a mix of gH and gH/gL. To assess this, we analyzed fractions from the leading and tailing edge of a gH/gL peak from size exclusion chromatography by SDS-PAGE (Fig. 1B). We observed differences in the electrophoretic mobility of gH, likely due to the presence of different glycoforms, and/or to proteolytic clipping, as evidenced by a band at ~35kDa in the later fractions that was not present in the early fraction (Fig. 1C). gL was present in comparable amounts in all fractions indicating that it was incorporated uniformly in our gH/gL preparation (Fig. C)".

Nevertheless, since we cannot completely rule out whether the purified protein is a mix of gH and gH/gL with this assay we changed the wording on lines 145-147 to say "Collectively, these analyses demonstrate that the recombinant KSHV gH/gL is glycosylated and capable of binding its cognate receptor".

We also note that in the processing of the new cryo-EM structures we include the in the revised manuscript, we do observe a population of particles that appear to lack gL and note this on lines 319-322: "We also observed a class of particles that appeared to lack gL (Fig. S5A) indicating that the co-transfection of gH and gL on separate plasmids did indeed result in a mixed pool of gH and gH/gL that we were unable to resolve by size exclusion chromatography (Fig.1B and C)."

Were there indications of particles lacking one domain (i.e., gL) in the 2D classes?
We did not observe any particles lacking one domain using nsEM, but we do by cryo-EM, which can achieve much higher resolution. Please see the above comment where we address this concern.

• Have the authors run AlphaFold multimer on gH/gL complexes with individual Fabs? This would be interesting and complementary information to the nsEM structural models reported in the manuscript. pTM and ipTM scores should be included to indicate the quality / confidence of the AF predicted models.

We ran the AF3 predictions as suggested, but they did not match the actual observed data. Thus, we will not include them in the paper, but they are provided here for the reviewer's information

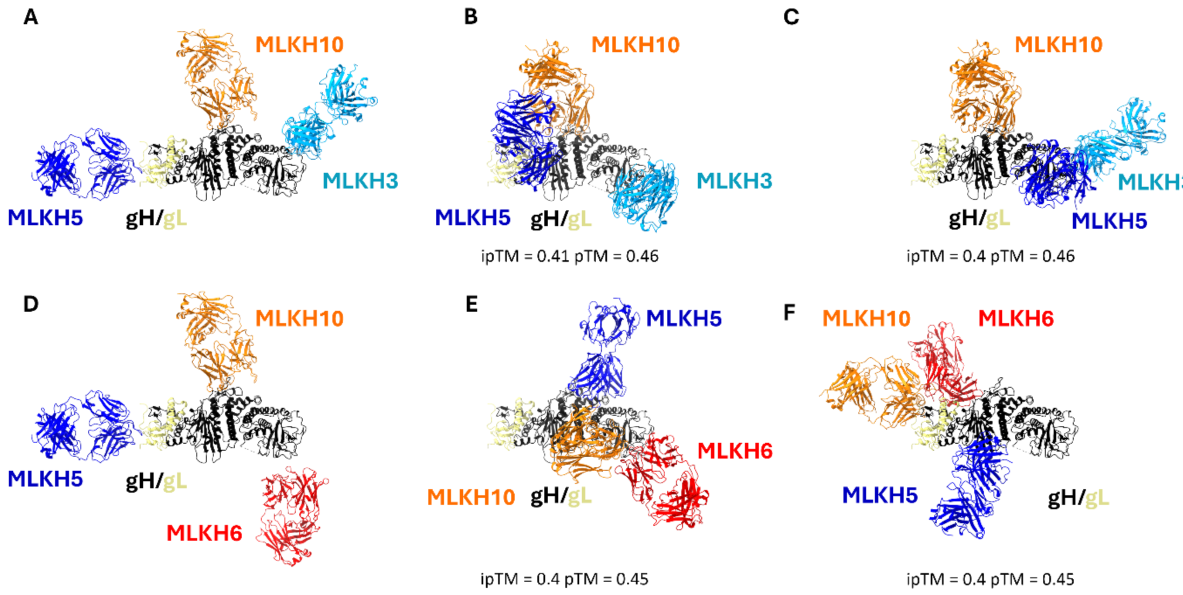

(A) The gH/gL/MLKH5/MLKH10/MLKH3 complex obtained from nsEM. (B and C) predictions of the same complex generated using AlphaFold 3. Two different AlphaFold 3 predictions are shown to emphasize the variation between the predictions and the lack of consensus. (D) The gH/gL/MLKH5/MLKH10/MLKH6 complex obtained from nsEM. (E and F) predictions of the same complex generated using AlphaFold 3. Two different AlphaFold 3 predictions are shown to emphasize the variation between the predictions and the lack of consensus. AF3 confidence scores are indicated for B-F.

Reviewer #2: Unclear whether necessary: If the data can be interpreted in a more careful way, the binding and neutralization experiments are good enough as is, otherwise some negative controls would be needed. If neutralization was done without heat inactivation, then a repeat with heat inactivation is needed to make statements about neutralization in Fig 2.

Binding and neutralization assays were performed with heat-inactivated plasma or serum. The results and methods on pages 632-633 have been updated to reflect this. We have also included additional presumed KSHV negative controls in Figures 2 and S2.

Part III – Minor Issues: Editorial and Data Presentation Modifications
Please use this section for editorial suggestions as well as relatively minor modifications of existing data that would enhance clarity.

Reviewer #1: • Fig 1 – D to F panels are not labelled

The panel labels have been corrected in the revised version of Figure 1.
• Line 57: consider including PMID: 38844783, PMID: 19536280 with reference 17

We added the latter reference since it is examining T cell responses in naturally infected humans. We opted not to include the first suggested reference since that study was done in humanized mice and changed the text of the manuscript on lines 67-68 to say "Natural KSHV infection elicits broad antibody[16] and T-cell[17, 18] responses."

• Line 80: references 34-38 do not all relate to the RGD motif being the binding site on integrins. The references should be dispereseed throughout the sentence to reflect corresctly what they refer to.

We revised this sentence to state that gB binds to integrins without specifically referring to the RBD motif (line 91).

• Line 86: consider adding PMID: 34499637 to reference 44

We have added this reference (now ref 46, line 97).
• Line 86: typo in "crystallography'

Corrected.

• Line 103: "12 high-affinity monoclonal ..."- high-affinity typically refers to Kd values in nM range, which was not the case for all 12 mAbs. Consider rephrasing.

Deleted "high-affinity".

• Line 103: "...map to 5 distinct epitope regions"- shouldn't it be 4? (Figure 7D)

In this case the 5 epitope regions refer to the 5 clusters identified in Figure 4, rather than the EM results which structurally characterize 4 of these clusters.

• Line 140: Could the authors clarify this statement? Was the blood collected and tested (Figure 2) prior to the RT-PCR that was used to assess the KSHV infection status?

For this analysis, detection of KSHV DNA by PCR was used to classify likely KSHV infection status. If KSHV was detected in the oral or blood specimens, we determined that the participant likely had established KSHV infection. If KSHV was not detected in oral or blood specimens, the participant may or may not have had KSHV infection. To increase the likelihood that KSHV PCR negative findings reflected a negative KSHV status, we utilized samples from young children (<1 yo) who were less likely to have acquired KSHV infection, as demonstrated by Gantt et al, who found no KSHV infection among Ugandan children under 1 years old (reference - https://doi.org/10.1093/infdis/jiw076-). It is possible, however, that some of these children had recently acquired KSHV infection and did not have detectable KSHV virus. We have not completed serology testing in this cohort and did not utilize serology to classify KSHV infection status in this study. We would likely have faced the same limitations with serology testing as we have with PCR testing, however, since a negative serology test could reflect early KSHV infection that has not yet mounted a detectable antibody response.

We addressed this on line 176-177 to make the statement clearer regarding KSHV infection status: "Weak neutralizing activity observed in these two participants may reflect early KSHV infection that was not detectable in blood or saliva by PCR testing"

• Lines 149 – 151: Could the authors hypolesize why those two samples (009 and 039-C1) could reduce infectivity by more than 50%?

Please see the response to the above point.

• Lines 163-172: If understood correctly, the sera from 3 KSHV+ and 1 KSHV- participants were used for isolation of B-cells. Does Figure 3B show results for one of the participants? It should be stated which one it was, because it seems that the majority of gH/gL specific B-cells (9 shown on Figure 3B) came from a single participant?

Fig 3B shows the staining from U35-022-MO, a PCR⁺ mother. This is now indicated in the legend of Figure 3B. We have also updated Table S3 to indicate the donor from whom the mAbs were isolated.

• Line 195: "… map to 6 epitope clusters…"Figure 4 shows presence of 5 clusters (the same was mentioned in the abstract, line 25).

Corrected.

• Line 289: It is confusing, but ephrin is the name for the ligand for Eph receptors. Therefore, 'ephrin' should be replaced with 'Eph'

"ephrin" changed to Eph.

• Line 301: 'intimates'was probably meant to be 'indicates'?

When used as a verb, intimates is appropriate.

• Lines 303-304: Could the authors speculate why competing mAbs do not have the same neutralization potency?

It is possible that mAbs that compete bind to an overlapping but not identical epitope on the protein, induce a conformational change, or otherwise block the mAbs ability to bind the protein without neutralizing on their own. We address these possibilities on lines 366-369 "High-resolution structures of neutralizing, and non-neutralizing mAbs in complex with gH/gL will help to discriminate whether the differences in neutralizing activity between competing mAbs is related to affinity, fine epitope specificities, or both".

• Line 311: an extra letter e is present

Removed.

• Line 319: 'ephrin A2' should be replaced with 'EphA2'

Corrected.

• Line 358, 551, 676: track changes kept on

Removed.

• Line 475: a space needed after the closed bracket

Corrected.

• Line 502: a definition of 'Endpoint titer' could be helpful for non-experts**.**

The definition of the endpoint titer has been added to the methods on lines 644-646.

• Lines 517-524: the word mouse should not be capitalized

Corrected.

• Lines 663-665: proof-reading is needed to correct wrongly capitalized words (in figure legends as well)

Grammatical errors in this section have been corrected.

• Line 697: the virus full-name (strain) should be included

Full strain name: rKSHV.219 added to text on line 885.

• Line 709: extra .

Corrected.

• Line 712: mAb instead of mab

Edited all instances of "mab" rather than "mAb."

• Line 719: word 'determined' should be replaced with a better one

Changed "determined" to "performed."

• Line 721: appoximating to 52ul of cells would be reasonable here

Changed to 52ul.

• References, lines 731-1014 are redundant (and missed a number somewhere) to the ones in lines 1110-1400, which seem to be correct.

Redundant references have been deleted.

• A table summarizing the findings would be helpful to present all the data in one place. For each mAb, there could be columns containing the information on human participant, binding affinity, assigned cluster, neutralization potency, ability to block cell-cell fusion, having AF model and / or experimental 3D reconstruction, GenBank number etc

We have included the requested table in the revised manuscript (S4).

• Figure 2A: consider marking the 2 participants whose PMBCs were used for B-cell sequencing. Instead of 'PCR+' and 'PCR-' labels, consider using 'KSHV+' and 'KSHV-'.

We prefer to use KSHV PCR^{+} and KSHV PCR^{-} since these samples were only tested based on PCR, not a diagnostic serological assay. It is however possible that they are in an early stage of infection and have not yet mounted a detectable antibody response.

• Line 1036: define what 'C1' refers to.

The legend has been corrected.

• Figure 3A: what are the 4 blue balls in the center representing?

We have revised Fig 3A to more clearly indicate what is streptavidin PE and what is gH/gL.

• Figure 5I: is there a special reason why the order of the mAbs (x axis) is not the same as on panels A, J and K?

We have revised the figure so that the mAbs are listed in the same order.

• Figure 7D: could Fabs MLKH6 and 12 be represented in different shades of red (or put one in grey and one in red) to distinguish them on the figure? Figure 7 in general is important, and merits a better representation of structures (for ex. maps more transparent, ribbon models more visible, panels larger).

Thanks for the suggestion. We updated Figure 7 to show MLKH12 in purple, which corresponds to it's Cluster 3 assignment. We have changed the color of MLKH12 in Figures 4-7 to match.

• Line 1100: '…fitted into the 'densities'. Strictly speaking, cryo-EM maps should not be referred to as densities but as Coulomb potential maps (PMID: 31400843).

Thank you for catching this. We note that these are negative stain EM data, not cryoEM. Therefore, we have changed "densities" to "ns-EM maps."

Reviewer #2: - Line 89: „binding" is missing.

Corrected.

- Line 135, line 418: Please explain RT-PCR. Why was reverse-transcriptase PCR used for KSHV diagnostics? What was the target? Where is the primer(probe?) set described with regard to the target? Ths information is missing from the manuscript.

We thank the author for catching this mistake. qPCR was used to detect KSHV DNA. We have clarified this on line 156. We have also added the following to the methods section Lines 621-625:"Blood and saliva samples were evaluated for KSHV DNA by a quantitative, high-throughput fluorescent-probe-based real-time polymerase chain reaction (PCR) assay (TaqMan assay, Applied Biosystems, Foster City, CA) of the ORF73 gene at the UCI-Fred Hutch Cancer Centre Laboratory in Kampala, Uganda as previously described[5,74]. Samples with >150 copies per mL of KSHV DNA were considered positive[75]."

- The authors constantly use either RMPI8226 or RPMI8226 throughout the text? Which is correct? Reference 55 is not easily accessible, RPMI8226 points to RRID:CVCL_0014 and makes sense.

Instances of the typographical "RMPI" have been edited to "RPMI".

- Fig 2 A and line 501: As per S1 Fig, there is a lot of binding to gH/gL below 1:100 dilution with the negative control serum. In fact, almost all PCR-negative children show endpoint titers below 100. This is a bit suspicious, could they in fact be negative? It is unclear from the methods how the threshold for the binding ELISA was defined (what were the control wells?). This needs to be made clear. Again, some negative controls might help here.

The methods section has been updated to clarify the definition of the endpoint titer. In the revised manuscript we now include additional control samples from a cohort of Seattle area donors with no known history of KSHV infection ("Presumed negative B-D." The serum from the donor included in the original manuscript is "Presumed negative A") to Figure 2, and show the raw ELISA data for all samples in S2. Given that none of the samples have been tested in a clinically validated serological assay, we hope the addition of these control data will allow for a more reasonable interpretation of our results.

Because our PCR negative grouping relies on clinical testing for KSHV, it is possible these PCR negative children were in fact recently infected and therefore have low levels of gH/gL antibodies but antibodies against other glycoproteins that are capable of neutralization. The text has been updated to reflect this on lines 176-177 "Weak neutralizing activity observed in these two participants (009-C1 and 039-C1) may be a reflection of early seroconversion that was not detected by the contemporaneous PCR testing of blood or oral swabs"

- Fig 2: Showing some controls would illustrative in particular for B, but also A. Were there any truly, or with high certainty, negative sera included? Other studies report rather low neutralization at 1:100, e.g. Byren et al, JGV 2024- At 1:50, Mortazavi et al, Viruses 2020 report similar neutralization, albeit with mostly KS patient seraa, and also not showing a negative control. Here, 1:10 was used, which is a very low dilution.

Please see the response to the above point regarding the inclusion of additional controls. We now note the differences observed here with other studies and suggest possible reasons (including testing of low serum dilution) on lines 420-428: "We detected serum neutralizing activity in nearly all mothers with PCR confirmed KSHV infection. This finding is at odds with studies that found that only ~10-20% of asymptomatic KSHV+ donors had KSHV neutralizing titers[21, 23], but in agreement with another study that found that neutralizing titers were higher in KSHV asymptomatic KSHV$^+$ participants as compared to participants with KS[22] or participants in remission from KS[23]. We note that we tested serum at a low dilution and used Vero target cells while the aforementioned studies used 293 cells. Thus, differences in the study populations, assays and serum dilutions tested could account for a lack of concordance across these studies".

- There is no mention of heat inactivation for the neutralization assay – this would be critical to distinguish complement-mediated effects from true neutralization. At 1:10, complement activation through binding antibodies will override any neutralization effects. It would also explain the high proportion of sera with neutralizing activity, also in PCR-negative children.

We apologize for this oversight. Serum or plasma was heat-inactivated at 56°C for 1 hour; the methods have been updated to reflect this on lines 632-633.

- Along those same lines: The overall fact that the mothers cohort universally has highly neutralizing serum activity is a bit suspicious compared to the literature. Kumar et al, PLoS One 2023, found that less than 10% of of asymptomatically infected individuals in a cohort from Zambia had neutralizing antibodies.

We note that we used a lower serum dilution 1:10 vs 1:50 in the Kumar study. We now explicitly state that we use a low serum dilution on line 168. We also discuss our findings relative to the Kumar study, and others please see response to the penultimate comment above.

- Line 243: „Collectively these data indicate that with the exception of MLKH 10 (Cluster 5), at least one mAb from each cluster can neutralize KSHV infection of epithelial cells, but

none can neutralize KSHV infection of B cells." This is quite an overinterpretation. The antibodies can't neutralize KSHV infection of RPMI8226 cells, that's it.

We agree and have clarified this statement on lines 278-281: "Collectively these data indicate that with the exception of MLKH 10 (Cluster 5), at least one mAb from each cluster can neutralize KSHV infection of epithelial cells, but none can neutralize KSHV infection of the RPMI8226 B cell line". We also note that in the discussion (lines 403-405): "further studies to evaluate the neutralizing activity of the gH/gL mAbs against KSHV infection of multiple B cell lines *in vitro* as well as in mice harboring susceptible human B cells are warranted."
